# Mindfulness is associated with improved psychological well-being but no change in stress biomarkers in breast cancer survivors with depression: a single group clinical pilot study

**DOI:** 10.1186/s12905-022-02116-y

**Published:** 2022-12-12

**Authors:** Silja Emilia Sakki, Heidi Marika Penttinen, Outi Maria Hilgert, Salla-Maarit Volanen, Tiina Saarto, Anu Raevuori

**Affiliations:** 1 Comprehensive Cancer Centre, Helsinki University Hospital, Helsinki, Finland; 2 Department of Oncology, Institute of Clinical Medicine, University of Helsinki, Helsinki, Finland; 3 Palliative Centre, Helsinki University Hospital, Helsinki, Finland; 4 Cancer Society of Pirkanmaa, Tampere, Finland; 5 Institute for Mindfulness in Medicine, Hemsbach, Germany; 6 Department of Public Health, Clinicum, University of Helsinki, Helsinki, Finland; 7 Folkhälsan Research Center, Helsinki, Finland; 8 Department of Psychiatry, Helsinki University Hospital, Helsinki, Finland

**Keywords:** Mindfulness, Mindfulness-based stress reduction, MBSR, Breast neoplasms, Breast cancer, Depression, Resilience, Biomarkers

## Abstract

**Background:**

The aim of this clinical single group pilot study was to assess mental well-being, psychological symptoms, and a set of stress biomarkers among breast cancer survivors with high depressive symptoms undergoing the Mindfulness-Based Stress Reduction (MBSR) program.

**Methods:**

Participants included 23 curatively treated breast cancer survivors from the Helsinki University Central Hospital with clinically significant symptoms of depression (Beck Depression Inventory > 13, and assessed by a psychiatrist), at 1-year post-operative follow-up. Mental wellbeing and psychological symptoms were assessed with self-reported questionnaires (Resilience Scale, Self-Compassion Scale, Five Facet Mindfulness Questionnaire, World Health Organization Quality of Life-questionnaire, Perceived Stress Scale, Beck Depression Inventory, Beck Anxiety Inventory, Insomnia Severity Index); in addition, stress response was assessed with biomarkers (cortisol, adrenocorticotropine, and high-sensitivity-CRP from blood; 24 h-cortisol from urine). All measures were addressed at baseline, mid-program (4 weeks after baseline) and at the completion of the 8-week MBSR program. Engagement to the independent mindfulness home practice was collected with a diary.

**Results:**

From baseline to the completion of the 8-week MBSR program, we observed significant increases in resilience (*d* = 0.60, *p* = 0.005), and quality of life (*d* = 0.92, *p* = 0.002), and significant reductions in symptoms of depression (*d* = − 1.17, *p* < 0.0001), anxiety (*d* = − 0.87, *p* < 0.0001), insomnia (*d* = − 0.58, *p* = 0.006), and marginally significant reduction in perceived stress (*d* = − 0.40, *p* = 0.09). We found no changes in self-compassion or mindfulness skills, nor in the stress biomarkers during or at the completion of the program. There was no association between the engagement time to the independent mindfulness practice and any of the outcomes.

**Conclusions:**

Attending the MBSR program was associated with increased wellbeing and reduced psychological symptoms in breast cancer survivors with clinically significant symptoms of depression. However, these favorably experienced changes did not transfer to the level of stress biomarkers during the 8-week program. Lack of association between the engagement in the mindfulness home practice and change in outcomes suggests that in the studied range of practice time, other qualities of MBSR despite the amount of independent practice may have a more important role for the improved wellbeing.

*Trial registration* ISRCTN12326308 at 16/02/2021, retrospectively registered.

**Supplementary Information:**

The online version contains supplementary material available at 10.1186/s12905-022-02116-y.

## Background

Every year over 2 million women worldwide get the diagnosis of breast cancer [1]. The treatment is curative in the majority of cases and approximately 90% of the patients are alive five years after receiving the diagnosis [2–4]. Long-term adverse effects of the treatment and quality of life have therefore become even more central issue in the field.

Especially during and shortly after the cancer treatment, women with breast cancer go through a challenging period. Many suffer from physical and psychological symptoms that affect their quality of life [5–8]. Impaired quality of life is often associated with menopausal symptoms, fatigue, and depression [9, 10]. According to a review, 40% of breast cancer survivors suffer from depressive symptoms within five years after breast cancer diagnosis [5]. Some suffer from condition resembling posttraumatic stress disorder that may last from months to several years [11]. Prevalence of depression in breast cancer survivors is shown to be the highest during the first year after the diagnosis [5, 8], and the risk remains elevated for more than five years [5]. Increased anxiety is also common [5, 12], but in long-term, no difference to the general population [5] in anxiety symptoms has been reported. Fear of cancer recurrence, physical ailments relating to cancer treatment, and challenges in returning to normal life have been reported to be the primary sources of distress [12]. For those in working-age, both physical and psychological symptoms affect the capability to return to work after the cancer treatment [6]. Health-care should provide rehabilitation for coping with these challenges.

Mindfulness refers to the awareness that arises from paying attention, on purpose, in the present moment, and non-judgmentally [13]. As a distinct from traditional medical approach, principles of mindfulness match to those of salutogenic approach that views mental disorders and mental wellbeing as two separate concepts [14], which together structure our mental health. A motivation for bringing mindfulness interventions in health care include expanding people’s sense of wellbeing and healing, despite illness symptoms and suffering that they may simultaneously have, and these programs have indeed been successful in enhancing recovery and the experience of wellness, wholeness, and a meaningful life in many patient groups [14, 15]. Among breast cancer survivors, mindfulness interventions have been shown to improve wide range of physical and psychological symptoms and quality of life [7, 8]. Specifically, in rehabilitation of breast cancer survivors, Mindfulness-Based Stress Reduction (MBSR) program has been associated with decreased depression and anxiety [7, 8], alleviation of fatigue [7], neuropathic pain [16], stress, and improved physical functioning and coping capacity, as well as posttraumatic growth [8]. Growing evidence also suggests that MBSR has favorable influence on immune system functions in women with breast cancer [8, 17].

Breast cancer survivors with longer time since the diagnosis and cancer treatments have shown more interest in mindfulness-based interventions compared to newly diagnosed patients [18]; together with elevated prevalence of depression in this population, their receptivity for mindfulness intervention is assumed to be high. Despite that several studies [19–22] suggest participants’ mean depression scores at baseline before the mindfulness intervention to be above the cut-off for at least mild depressive symptoms, study inclusion criteria have only rarely included cut-off for depression symptoms [20]. Yet alleviation of depression, anxiety, and related symptoms in this population including both those with and without increased depressive symptoms has been shown robustly [23, 24]. In turn, there are few or no studies that examine resilience [25], mindfulness skills [26], and self-compassion, in women with breast cancer. Relatively little is also known about the relationship between mindfulness practice time and different outcomes in participants with or in recovery of cancer, or cancer survivors [27], and the same applies to mindfulness-based interventions and changes of objective markers of stress in breast-cancer survivors, particularly among those with depression [28].

The aim of this study was therefore to assess changes in mental wellbeing utilizing instruments of positive psychology, and psychological symptoms, and extensive set of stress biomarkers associated with physiological stress response among breast cancer survivors with high depressive symptoms, who underwent the MBSR program. A single-group, clinical pilot study design was selected due to local circumstances, i.e. lack of previous studies of mindfulness interventions in Finnish university hospital patients.

## Methods

### Study design

This pilot study utilized a single-group pretest and repeated mid-, posttest experimental design with systematic sampling. All curatively treated, disease-free consecutive patients before their one-year follow-up were sampled for four months period. The goal was to assess the changes in mental well-being, psychological symptoms, and stress biomarker levels in breast cancer survivors with increased symptoms of depression who participated 8-week structured MBSR program. Outcome variables were measured at three time points: at baseline; mid-intervention, i.e. 4-weeks after baseline in order to track potential changes in the outcomes during the intervention; and immediately post-intervention, i.e. 8-weeks after the baseline. Background characteristics including menopausal status, employment, social support, and other diseases were collected with a questionnaire at baseline. Details related to the breast tumor and surgery, as well as adjuvant treatment were extracted from the participants medical records. Biomarkers of stress response were sampled in the hospital laboratory using standardized methods.

The trial is retrospectively registered with ISRCTN registry (ISRCTN12326308, accessible at https://www.isrctn.com/ISRCTN12326308). Approval for the study was provided by the Ethics Committee of the Hospital District of Helsinki and Uusimaa (TMK03, 60/13/03/03/2016). Patient safety was monitored by systematic monitoring of adverse/ serious adverse events by general practitioner and MBSR instructor (O.H.) and a psychiatrist (A.R.) of the team.

### Participants and recruitment

Participants included female breast cancer survivors with operated invasive breast cancer (T1-4 N0-3 M0) one year earlier, treated with adjuvant chemotherapy, endocrine therapy and/or radiotherapy. They were identified from the database of The Comprehensive Cancer Centre of Helsinki University Central Hospital. Screening letter including information of the study, self-reported assessment of depressive symptoms, and preliminary informed consent was sent to 217 curatively treated and disease-free consecutive patients in the Comprehensive Cancer Centre of Helsinki University Central Hospital before their one-year follow-up appointment between January 2017 and April 2017. To cover working age women, participants were aged 25–65 years. Exclusion criteria included metastatic disease, lack of motivation to attend, substance abuse, suicidality, psychotic symptoms, untreated posttraumatic stress disorder, or severe social phobia. The study had no effect on the participants’ standard medical care.

**Fig. 1:**
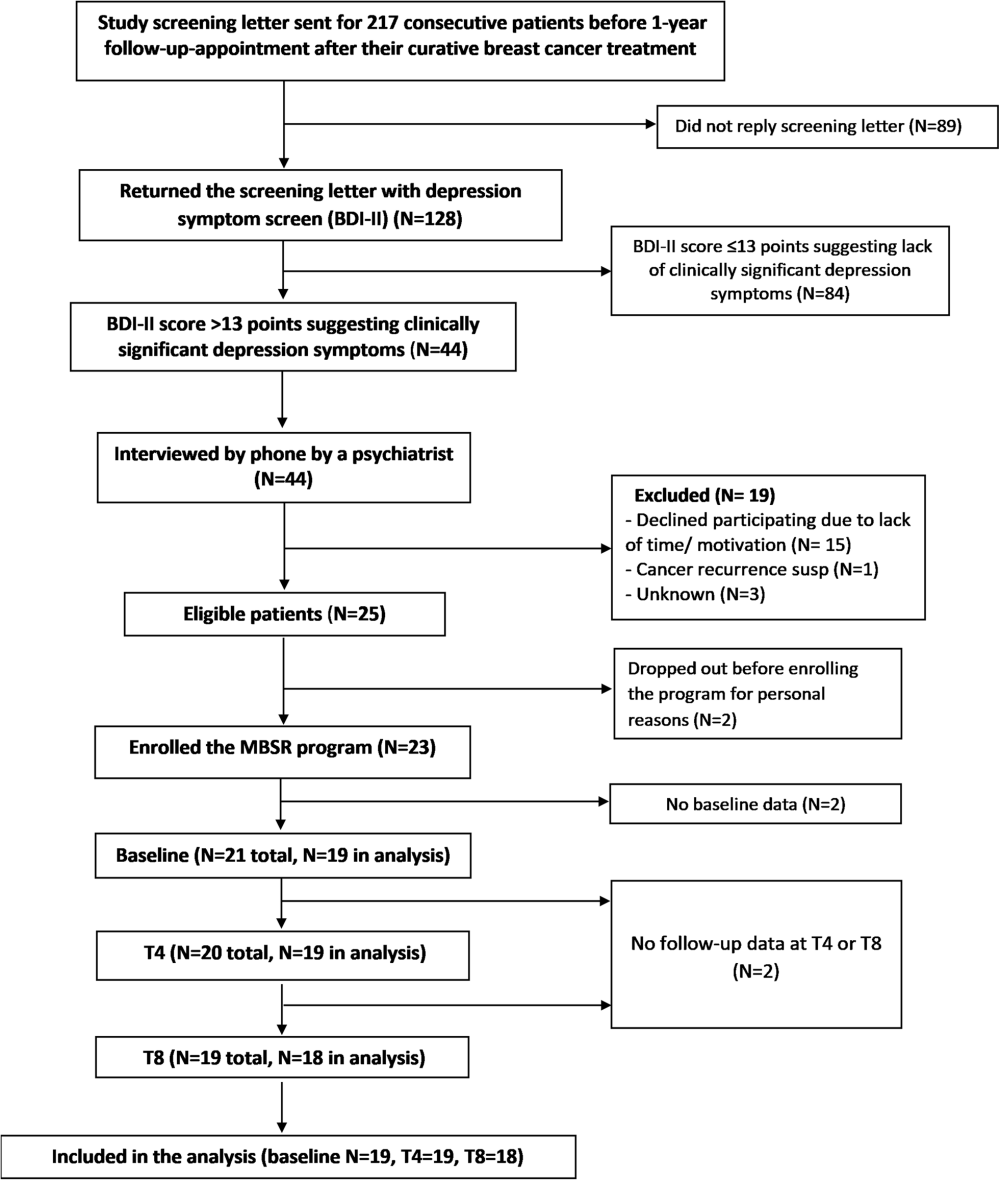
CONSORT Flow diagram

Of women who received the screening letter, 128 (59%) showed interest in the pilot study by returning the BDI questionnaire, and 34% (N = 44) had BDI-II score > 13 points suggesting clinically significant depression symptoms. They were interviewed by phone by a psychiatrist (A.R.) to assess the eligibility, including clinical significance of the depression symptoms. The first 25 eligible patients were recruited, and signed the written informed consent for the study; of them, 23 started the program. The flow chart of the study is presented in Fig. 1.

### Intervention

MBSR is a manualized, group-based 8-week program developed for systematic training for mindfulness skills in and between the sessions [13]. The program consists of eight weekly group sessions (each lasting 2.5 h in the current study), one day long silent retreat, and mindfulness home practice between the sessions (recommendation 45 min daily). The weekly sessions include standardized elements consisting of different mental and physical mindfulness practices: (1) body-scan practice, (2) mental practice focusing one's attention on the breath, (3) mindful movements and yoga practice, and (4) practicing being fully aware during everyday activities by using the breath as an anchor for the attention. Between-session practice consists of formal (such as body-scan, focusing on one’s breath, mindful movements) and informal mindfulness home practice (being fully aware during everyday activities) that trains attention and develops the ability to respond to mental and physical experiences. Between-session mindfulness practice is considered an essential element of the program, and it is needed for developing an accepting, non-reactive attitude and mindful awareness in each moment [15]. Participants underwent the program between August 30th and October 18th in 2017. It was led by a general practitioner and a certified MBSR-instructor (O.H.).

### Measures

#### Resilience

Resilience describes psychological adaptation ability in the face of adversity. It was measured with Resilience Scale [29] containing 25 items scoring 1–7, and total scores ranging 25–175. Scores 25–100 suggest very low; 101–115 low resilience; 116–130 suggest on the low end resilience; 131–140 moderate, 146–160 moderately high and 161–175 high resilience. The Resilience Scale has demonstrated good internal consistency with Cronbach’s alpha ranging 0.76–0.91 [29].

#### Self-compassion

Self-compassion was assessed by Self-compassion Scale (short form), SCS-SF [30]. It contains 12 items rated 1–5, higher scores representing more self-compassion, and total scores ranging from 12 to 60. Sub-scales cover self-kindness, self-judgement, common humanity, isolation, mindfulness and over-identification. The internal consistency is reported high with Cronbach alpha of 0.86 [30].

#### Mindfulness skills

Adopting mindfulness skills were evaluated with Five Facet Mindfulness Questionnaire (FFMQ) [31]. It covers five aspects of mindfulness: observing, describing, acting with awareness, non-judging of inner experience, and non-reactivity to inner experience. The questionnaire contains 24 items rated 1–5, total scores ranging between 24 and 120, and higher scores representing higher mindfulness skills. Internal consistency of FFMQ is shown to be good with Cronbach alpha ranging 0.77–0.93 [32].

#### Psychological stress

Perceived Stress scale, PSS-10 [33], was used to measure psychological stress. PSS-10 has 10 items each rated 0–4, with total score ranging between 0 and 40. Scores 0–13 suggest low, 14–26 moderate, and 27–40 high level of perceived stress. PSS-10 has demonstrated good reliability with Cronbach’s alpha ranging 0.78–0.91 [34].

#### Quality of life

World Health Organization Quality of Life WHOQOL-BREF–questionnaire [35] was used to measure overall quality of life. It contains 26 items measuring four domains: physical health, psychological health, social relationships and environment. For each domain the scoring is 0–100 points, and total score is between 0 and 400 points. Internal consistency of the instrument is good, with Cronbach’s alpha ranging from 0.66 (domain 3) to 0.84 (domain 1) [35].

#### Symptoms of depression

Depressive symptoms were measured by Beck Depression Inventory-II (BDI I-II) [36]. The questionnaire contains 21 items rated 0–3 points, with total score ranging between 0 and 63. Scores of 14–19 suggest mild depression; 20–28 moderate depression, and 29–63 severe depression. The BDI-II has demonstrated high internal consistency with Cronbach’s alpha of 0.9 [37].

#### Symptoms of anxiety

Beck Anxiety Inventory, BAI [38], was used to assess anxiety symptoms. The questionnaire has 21 items rated 0–3, with total score ranging from 0 to 63. Scores 10–18 suggest mild to moderate; 19–29 moderate to moderately severe and 30–63 severe anxiety. The internal consistency of BAI is reported high with Cronbach’s alpha ranging 0.90–0.94 [39].

#### Insomnia

Nighttime and daytime components of insomnia were measured by Insomnia Severity Index, ISI [40]. It is a 7-item self-report questionnaire with items rated between 0 and 4, total score ranging from 0 to 28. Scores 8–14 suggest sub-threshold insomnia; 15–21 clinical insomnia of moderate severity, and scores 22–28 clinically severe insomnia. ISI has demonstrated high internal consistency, with Cronbach’s alpha ranging 0.90–0.91 [40].

#### Stress biomarkers

The laboratory tests were obtained to measure the level of bodily stress and level of chronic inflammation. The samples included high-sensitivity C-reactive protein from the serum (S-hs-CRP, normal values for women 0.05–3), serum cortisol (S-Cortisol, normal values for adults 150–650 nmol/l), plasma adrenocorticotropin (P-ACTH, normal value below 46 ng/l), and 24-h cortisol from urine (dU-Cortisol, normal value 30–144 nmol/24 h) [41].

#### The independent mindfulness practice

The participants filled in a diary of the independent mindfulness practice they conducted at home. They recorded daily minutes of formal mindfulness meditations, such as sitting or body scanning, and informal mindfulness practices of everyday life during the 8-week MBSR program (56 days in total). Participants’ formal practices were supported by audio recordings provided by the program instructor. In these, participants were given guidance to the nature and content of the practice, e.g. suggestions of the posture adopted and how attention is directed. Informal practices were addressed to cultivate mindfulness in everyday life, such as by focusing awareness on everyday activities and pleasant or unpleasant experiences. The total time of mindfulness practice included the sum of formal and informal practice in minutes per day.

### Statistical analysis

Participants with baseline measurement and at least one follow-up were included in a longitudinal analysis. In similar manner, a sensitivity analysis using intention to treat approach was conducted including all participants with data from any (at least one) time point. The changes in the self-reported questionnaire scores and in the biomarker values between baseline, 4-week and 8-week follow-ups were analyzed with repeated measures analysis of variance (ANOVA). The 4-week and 8-week follow-up scores were compared to baseline scores using Dunnett’s adjustment in pairwise comparisons in repeated measures ANOVA model. Unstructured covariance structure was used in models to account for the correlation between repeated measurements. Restricted maximum likelihood estimation method was used to allow for missing values in the follow-up measurements. Due to skewed distributions, hs-CRP, P-ACTH and dU-Cortisol values were natural log-transformed for statistical analysis. The effect sizes of the change from baseline to mid-intervention, and from baseline to intervention completion were calculated by *Cohen's d* using pooled standard deviation at the baseline. Effect sizes of 0.2 were considered as small, 0.5 as medium, and 0.8 as large. The association between the quantity of mindfulness practice and the 8-week change in the self-reported questionnaire scores was examined using Pearson correlation coefficients. *p*-values < 0.05 were considered statistically significant in two-sided tests. Statistical analyses were done with SAS System for Windows, version 9.4 (SAS Institute Inc., Cary, NC).

## Results

### Participant characteristics

At baseline, 23 participants started the MBSR program. Of them, three (13%) individuals dropped out during the program, while 19 individuals provided data from baseline and at least one of the follow-ups, and were included in the analyses (Fig. 1); in addition, sensitivity analysis included all participants (N = 21) who provided data in at least one time point. All participants were Caucasian women (mean age 55 years). For participant characteristics, see Table 1.

**Table 1:** Participant characteristics at baseline

	N
Participants, total	23
Age, range (mean)	38–64y (55y)
Menopausal status	
Premenopausal	8 (35%)
Postmenopausal	15 (65%)
Employment	
Self-employed	3 (13%)
Upper-level employees	4 (17%)
Lower-level employees	6 (26%)
Manual workers	1 (4%)
Students	0 (0%)
Pensioners, housewives	3 (13%)
Unemployed	2 (9%)
No data	4 (17%)
Social support	
Poor	2 (9%)
Moderate	11 (48%)
Good	8 (35%)
No data	2 (9%)
TNM (T = tumor size, N = number of metastatic regional lymphnodes)	
T1(≤ 20 mm)	14 (61%)
T2(21–50 mm)	5 (22%)
T (> 50 mm)	2 (9%)
T4 (any size with extension to chest wall or skin)	0 (0%)
DCIS (ductal carcinoma in situ)	2 (9%)
N0(0)	12 (52%)
N1(1–3)	8 (35%)
N2(4–9)	3 (13%)
N3(> 9)	0 (0%)
Breast surgery	
Mastectomy	9 (39%)
Conserving	14 (61%)
Adjuvant treatment	
Chemotherapy	15 (65%)
Anti-HER2-therapy	1 (4%)
Radiotherapy	20 (87%)
Endocrine treatment	17 (74%)
Other diseases	
Hypertension	6 (26%)
Cardiac disease	1 (4%)
Diabetes	2 (9%)
Asthma	4 (17%)
Hypothyreosis	1 (4%)
Allergies	4 (17%)
Arthrosis	4 (17%)
Other reported	6 (26%)

### Independent mindfulness practice

The mean daily minutes of total (formal plus informal) mindfulness home practice was 47 (SD 14, median 46, range 23–77 min) among those 15 participants who practiced > 0 min. Among them, mean daily minutes of the formal mindfulness home practice was 39 (SD 8, median 40, range 23–53 min), and mean daily minutes of the informal mindfulness home practice was 8 (SD 9, median 3, range 0–32 min). Associations between the quantity of mindfulness home practice and the change in any of the measures were not significant (analyses included participants reporting > 0 min of any independent home practice) (Table 2).

**Table 2:** Pearson correlations between the mindfulness practice time and 8-week change in the questionnaire scores

	Resilience Scale	Five Facet Mindfulness Questionnaire (FFMQ)	Self-Compassion Scale (short form) SSF-SF	Perceived Stress Scale (PSS)	WHO Quality of Life (WHOQOL-BREF)	Beck Depression Inventory (BDI)	Beck Anxiety Inventory (BAI)	Insomnia Severity Index (ISI)
Independent home practise, all	0.15	0.06	− 0.29	− 0.02	0.27	− 0.04	− 0.02	− 0.12
Formal practise	− 0.17	0.21	− 0.03	− 0.10	0.21	0.12	− 0.10	0.28
Informal practise	0.37	− 0.10	− 0.40	0.05	0.22	− 0.17	0.05	− 0.42

Participants who practiced at home > 0 min per day (N = 15) were taken into account in the analysis

### Psychological symptoms, mental wellbeing and stress biomarkers

From baseline to the completion (8 weeks) of the MBSR program, there was a significant increase in resilience (Resilience Scale: mean change 6.5, 95% Confidence Interval [CI] 0.9 to 12.1, Cohen’s *d* = 0.60, *p* = 0.005), and the quality of life (WHOQOL-BREF: mean change 6.0, 95% CI 2.0 to 10.0, Cohen’s *d* = 0.92, *p* = 0.002). Likewise, from baseline to the completion of the program, there was a significant reduction in symptoms of depression (BDI-II: mean change − 6.1, 95% CI − 9.0 to − 3.3, Cohen’s *d* = − 1.17, *p* < 0.0001), anxiety (BAI: mean change − 4.7, 95% CI − 7.2 to − 2.2, Cohen’s *d* = − 0.87, *p* < 0.0001), and insomnia (ISI: mean change − 3.3, 95% CI − 5.5 to − 1.2, Cohen’s *d* = − 0.58, *p* = 0.006); reduction in perceived stress was marginally significant (PSS: mean change − 1.2, 95% CI − 2.6 to 0.1 Cohen’s *d* = − 0.40, *p* = 0.09). From baseline to the mid-program (4 weeks), none of the changes above were significant. No changes in self-compassion or mindfulness skills were observed from baseline to mid-program or to the program completion (Self-Compassion Scale: mean change 0.3, 95% CI − 1.4 to 2.0, Cohen’s *d* = 0.09, *p* = 0.35; Five Facet Mindfulness Questionnaire: mean change − 0.6, 95% CI − 3.6 to 2.3, Cohen’s *d* = − 0.9, *p* = 0.41) (Table 3). Results from the sensitivity analysis (intent-to-treat analysis) were essentially consistent with the results above (Additional file: Table S1) with significant changes in resilience, quality of life, depression and anxiety symptoms, and insomnia; marginally significant change in perceived stress, and no changes in self-compassion and mindfulness skills. Analysis showed no changes in any of the biomarkers of stress from baseline to the mid-program or to the program completion (8 weeks) (Table 4).

**Table 3:** Changes in the self-reported questionnaire scores during the 8-week MBSR-program (Mindfulness-Based Stress Reduction)

Measure	Time	N	Mean score (SD)	Mean change (95% CI) compared to baseline	Effect size, Cohen’s *d*	T-statistics	*p*-value*	F-statistics	Global *p*-value for time effect
Resilience Scale	Baseline	19	86.3 (10.8)	–			–		0.005
	4 weeks	18	85.1 (13.5)	− 0.9 (− 7.2 to 5.5)	− 0.08	− 0.32	0.92	7.38	
	8 weeks	17	92.8 (11.7)	6.5 (0.9 to 12.1)	0.60	2.78	0.02		
Self-Compassion Scale (short form) SCS-SF	Baseline	18	38.9 (3.4)	–			–		0.35
	4 weeks	17	40.0 (3.0)	1.1 (− 0.7 to 2.8)	0.31	1.43	0.28	1.11	
	8 weeks	16	39.3 (3.9)	0.3 (− 1.4 to 2.0)	0.09	0.41	0.88		
Five Facet Mindfulness Questionnaire (FFMQ)	Baseline	19	74.1 (7.0)	–			–		0.41
	4 weeks	17	75.2 (6.9)	0.7 (− 2.9 to 4.3)	0.10	0.45	0.83	0.93	
	8 weeks	17	73.4 (4.6)	− 0.6 (− 3.6 to 2.3)	− 0.09	− 0.51	0.80		
Perceived Stress Scale (PSS)	Baseline	19	20.9 (3.1)	–			–		0.09
	4 weeks	18	21.2 (3.0)	0.3 (− 0.9 to 1.6)	0.10	0.62	0.79	2.76	
	8 weeks	17	19.6 (3.7)	− 1.2 (− 2.6 to 0.1)	− 0.40	− 2.18	0.08		
WHO Quality of Life (WHOQOL-BREF)	Baseline	19	82.5 (6.5)	–			–		0.002
	4 weeks	18	83.9 (13.0)	2.4 (− 2.6 to 7.4)	0.37	1.11	0.4	8.77	
	8 weeks	17	88.7 (10.1)	6.0 (2.0 to 10.0)	0.92	3.48	0.005		
Beck Depression Inventory (BDI)	Baseline	19	16.1 (5.2)	–			–		< 0.0001
	4 weeks	17	14.1 (6.3)	− 2.2 (− 5.6 to 1.1)	− 0.43	− 1.55	0.2	22.05	
	8 weeks	17	9.6 (4.6)	− 6.1 (− 9.0 to − 3.3)	− 1.17	− 5.04	0.0002		
Beck Anxiety Inventory (BAI)	Baseline	19	12.4 (5.4)	–			–		< 0.0001
	4 weeks	18	11.9 (5.2)	− 0.6 (− 2.7 to 1.6)	− 0.10	− 0.62	0.71	20.16	
	8 weeks	17	7.5 (4.0)	− 4.7 (− 7.2 to − 2.2)	− 0.87	− 4.49	0.0006		
Insomnia Severity Index (ISI)	Baseline	19	11.7 (5.7)	–			–		0.006
	4 weeks	18	10.4 (5.4)	− 1.5 (− 3.5 to 0.6)	− 0.25	− 1.69	0.19	6.99	
	8 weeks	17	8.8 (5.1)	− 3.3 (− 5.5 to − 1.2)	− 0.58	− 3.71	0.0033		

*SD* Standard Deviation, *CI* Confidence Interval

*p-value for mean change at 4 and 8 weeks compared to the baseline (Dunnett`s adjustment method); repeated measures analysis of variance

**Table 4:** Changes in the biomarker values during the 8-week MBSR-program (Mindfulness-Based Stress Reduction)

Biomarker		N	Mean values	Mean change compared to baseline (95% CI)	Effect size, Cohen’s *d*	T-statistics	*p*-value*	F-statistics	*p*-value, global
hs-CRP† (mg/l)	Baseline	19	0.21	**–**			**–**		0.49
	4 weeks	18	0.41	0.18 (− 0.24 to 0.60)	0.20	1.03	0.52	0.75	
	8 weeks	18	0.36	0.18 (− 0.28 to 0.61)	0.19	0.90	0.60		
Plasma-ACTH† (ng/l)	Baseline	18	2.79	–			–		0.19
	4 weeks	17	2.62	− 0.16 (− 0.51 to 0.19)	− 0.26	− 1.08	0.43	1.85	
	8 weeks	17	2.86	0.025 (− 0.27 to 0.37)	0.08	0.36	0.89		
Serum-Cortisol (nmol/l)	Baseline	19	361.2	–			–		0.93
	4 weeks	19	364.8	3.6 (− 58.9 to 66.2)	0.03	0.14	0.98	0.08	
	8 weeks	18	359.0	− 6.4 (− 80.8 to 68.0)	− 0.05	− 0.21	0.97		
24-h-Urine-Cortisol† (nmol/24 h)	Baseline	18	4.04	–			–		0.89
	4 weeks	18	4.03	− 0.01 (− 0.47 to 0.45)	− 0.02	− 0.06	1.00	0.12	
	8 weeks	15	4.08	0.07 (− 0.38 to 0.52)	0.12	0.37	0.90		

*ACTH* Adrenocorticotropine*, hs-CRP* High-sensitivity, C-reactive Protein, *CI* Confidence Interval

*p-value for mean change at 4 and 8 weeks compared to the baseline (Dunnett`s adjustment method); repeated measures analysis of variance

^†^hs-CRP, ACTH and dU-Cortisol values were natural log-transformed for statistical analysis

## Discussion

This study focused on an important clinical population, breast cancer survivors with significant depressive symptoms. The results suggest that among these women, 8-week MBSR program was associated with increased psychological resilience and quality of life, and with a trend for decreased experience of stress; and reduced symptoms of depression, anxiety, and insomnia. These findings implicate that women attending the MBSR program attained qualities that are important for overall mental wellbeing, and for dealing effectively with their experiences with cancer and depression, as well as for protection of re-emerging depression. The increase in psychological well-being did however not transfer to the level of biomarkers in the course of the 8-week program. We cannot therefore state that attending MBSR program would be associated with favorable changes in physical health among breast cancer survivors within the studied time frame. We neither observed associations with the improved well-being and participants’ engagement to the mindfulness home practice. This suggests that in the studied range of practice time, other qualities of MBSR despite the amount of independent mindfulness practice may have a more important role for the improved wellbeing.

Of the qualities of positive psychology, improved quality of life [23, 42], coping capacity [42], and posttraumatic growth [8] have been reported in earlier studies among women with breast cancer. To these, our study adds improved resilience; and self-compassion and mindfulness skills, which however remained unchanged. Resilience has been defined as an ability to ‘bounce back’ from adversity combined with awareness of one’s own internal coherence [43]. As a consequence, resilience provides wide-ranging benefits when an individual faces challenging life events and suffering, such as severe illness. Interestingly, breast cancer survivors with higher resilience have been shown to exhibit weaker associations between stress and depressive symptoms, and weaker association between stress and inflammation-associated depressive symptoms, specifically [25]. The authors suggested that psychosocial resilience may play an important role in protecting individuals from inflammation-associated depressive symptoms under conditions of high stress [25]. In a biological level, psychosocial stressors have been shown to produce inflammation [44], which in turn elicits sickness behaviors that includes depressive symptoms [45]; likewise, resilience can be viewed as including complex processes that require integration of multiple central (such as hypothalamic–pituitary–adrenal-axis [HPA-axis]) and peripheral systems (such as immune system and gut microbiota) [46]. Together these influences highlight the interconnectedness of the mind and body, and suggest that holistic interventions taking biopsychosocial factors into account among breast cancer survivors are important.

Reason for the lack of change in self-compassion and mindfulness skills in our study is unclear, as MBSR program encourages cultivating compassion towards oneself and others, as well as general mindful way of being. It is possible that the participants had difficulties in recognizing and verbalizing their mindfulness-associated capacity in the questionnaire. On the other hand, we neither observed association between the independent mindfulness practice and the improvement of psychological symptoms. This suggests that other factors related to participating the MBSR program, despite developing mindful awareness, such as group support and interaction with the instructor, had a more important role for improved psychological well-being.

The impact of the engagement to mindfulness home practice has mostly been studied in non-cancer populations [27], among whom continuing practice after the mindfulness intervention has been shown to prevent relapse of recurrent depression [47]. A meta-analysis of 28 studies showed an association (r = 0.26, 95% CI 0.19–0.34) between engaging in home practice (mean 30 min daily) and unspecified psychological and physical treatment outcomes [27]. The significant association held across (predominantly non-cancer) clinical and nonclinical participant groups. However, in line with our study, a pilot study with breast and prostate cancer survivors did not report an association between home mindfulness practice and improving psychological stress or quality of life; nor with immune parameters during the MBSR program or at one-year follow-up [48]. The authors suggested that further home practice was perhaps not necessary for improved wellbeing, which was already achieved during the program and was considered self-sustaining. We are unable to determine whether the same explanation might apply to our study. Participants of our study engaged in the home practice diligently; 53% engaged over the recommended 45 min per day, and almost all (93%) of those doing any home practice, did it for over 30 min a day. In case there was a threshold-effect of the amount of home practice time and symptom change, our study could not detect it due to small number of those practicing little or not at all (N = 4).

Growing evidence suggests that mindfulness has favorable influence on immune system function among breast cancer patients [8, 42, 49, 50]. Beneficial changes in NK cell activity [8, 42], cytokine levels [42, 48], and number of NK- and B-cells [8] have been reported among participants during the MBSR program. There are also few reports of the association of attending the mindfulness intervention and cortisol levels: among recently diagnosed breast cancer patients, evening plasma cortisol levels were significantly lower in those attending the MBSR at the end of the program compared to control individuals [42]. In addition, decrease in saliva cortisol [48, 50] and IL6 [49] has been reported among recovering patients with breast cancer after up to one year attending the MBSR program [48]. The biomarkers of stress measured in our study, sera and extensive 24-h-urine cortisol, ACTH and hs-CRP, did not change during the 8-week MBSR program. A plausible explanation could be lack of follow-up after the program and/or hypoactivation of stress response, given the significant symptoms of clinical depression among all participants. In depressed women, disruptions in HPA-axis are also shown to be associated with dysregulation of hypothalamic-pituitary–gonadal (HPG)-axis [51], likewise present in study participants. In some studies, changes in cytokine levels among breast and prostate cancer survivors have been reported appearing only in longer follow-ups of 4 weeks [17], 12 weeks [49] or 6–12 months [48]. It has also been shown that stress biomarkers act differently during or shortly after breast cancer treatment, when psychological and physical stress levels are higher [42] compared to the longer follow-up [48]. Compared to our study, the vast majority of participants in above mentioned studies were at earlier stage of recovery [8, 42, 49], did not have as distinct symptoms of depression, or the follow-up after the mindfulness intervention lasted longer allowing more lengthy influence on the stress biomarkers, which may contribute to the conflicting findings. Due to the diurnal and individual variation of the cortisol levels, the array of cortisol-related stress biomarkers including 24-h-urine cortisol in our study were comprehensive indicators compared to those in the other studies, to track changes in the stress-response, if they existed.

Strengths of the study include participants with clinically significant depression symptoms at the study inclusion, measures of stress biomarkers collected from blood and urine samples, self-reported questionnaire data including measures of positive psychology previously understudied in this patient population, and information of the time of the independent mindfulness home practice. Participants’ drop-out was moderate. Limitations include small sample and uncontrolled design, which prevents causal inferences regarding the MBSR program and outcome changes. Reliability measures for the presented data were not analyzed. Fidelity to the intervention protocol was tracked with a dairy of the mindfulness home practice held by each participant, but not with the overall attendance rates for 8 MBSR sessions. Participants’ potential comorbid disease, such as diabetes or autoimmune conditions, based only on self-report; they may have had an impact on stress and inflammation biomarkers, but comorbid conditions were not taken into account in the analyses. Of measures, World Health Organization Quality of Life WHOQOL-BREF–questionnaire was used instead of cancer specific quality of life measures. For mindfulness skills, total score of FFMQ was used despite some validation studies [52] suggesting that one of the five facets, Observing subscale, may not function optimally among mindfulness naïve individuals, such as participants in our study. Lack of follow-up after the MBSR program prevented us from assessing how the observed outcomes would have evolved over time. Future studies should invest in controlled designs with long follow-up periods.

## Conclusions

Results of this study suggest that among working-age breast cancer survivors with clinically significant depressive symptoms, attending mindfulness-program was associated with increased resilience. In this population, attending mindfulness program appears to be associated with psychological qualities that support general mental health and wellbeing, and mitigate the vulnerability to distressing current and future life experiences. Our results are in line with previous reports in that mindfulness-program was associated with decreased depression and anxiety symptoms, insomnia, psychological stress and with improved quality of life. These favorably experienced changes did however not transfer to the level of stress biomarkers during the 8-week program. Lack of association between the engagement in the mindfulness home practice and change in outcomes suggests that in the studied range of practice time, other qualities of MBSR, such as group support, may have a more important role for the improved wellbeing.

## Supplementary Information

**Additional file 1: Table S1.** Results from the sensitivity analysis (Intention To Treat): self-reported questionnaire scores during the MBSR-program.

**Additional file 2: Table S2.** Dataset for the study.

## Data Availability

Dataset analysed in this study are included in this published article and additional file (Additional file 1: Table S2).
