# Supplementary material for: Mindfulness is associated with improved psychological well-being but no change in stress biomarkers in breast cancer survivors with depression: a single group clinical pilot study
Source: BMC Womens Health. 2022 Dec 12;22:518. doi: 10.1186/s12905-022-02116-y (PMC9743600; doi:10.1186/s12905-022-02116-y)
Supplement: Supplementary file 1 — Additional file 1: Table S1. Results from the sensitivity analysis (Intention To Treat): self-reported questionnaire scores during the MBSR-program. [file 12905_2022_2116_MOESM1_ESM.docx]

**Additional table 1.** Results from the sensitivity analysis (Intention To Treat): self-reported questionnaire scores during the MBSR-program.

| **Measure** |  | **N** | **Mean score (SD)** | **Mean change (95% CI) compared to baseline** | **Effect size, Cohen’s *d*** | **p-value*** | **p-value, global** |
| --- | --- | --- | --- | --- | --- | --- | --- |
| **Resilience Scale** | Baseline | 21 | 86.2 (10.5) | - |  | - | 0.003 |
|  | 4 weeks | 20 | 85.3 (13.4) | -0.2 (-6.2 to 5.9) | -0.02 | 0.996 |  |
|  | 8 weeks | 19 | 92.7 (11.1) | 6.7 (1.4 to 12.0) | 0.64 | 0.01 |  |
| **Self-Compassion Scale (short form) SCS-SF** | Baseline | 20 | 38.1 (3.2) | - |  | - | 0.16 |
|  | 4 weeks | 20 | 40.1 (3.4) | 1.3 (-0.5 to 3.1) | 0.41 | 0.17 |  |
|  | 8 weeks | 19 | 38.8 (4.3) | -0.1 (-1.8 to 1.7) | -0.03 | 0.996 |  |
| **Five Facet Mindfulness Questionnaire (FFMQ)** | Baseline | 21 | 74.1 (6.6) | - |  | - | 0.37 |
|  | 4 weeks | 19 | 74.3 (7.0) | 0.5 (-2.9 to 3.9) | 0.08 | 0.89 |  |
|  | 8 weeks | 19 | 72.8 (4.8) | -0.8 (-3.6 to 2.0) | -0.12 | 0.69 |  |
| **Perceived Stress Scale (PSS)** | Baseline | 21 | 21.1 (3.1) | - |  | - | 0.08 |
|  | 4 weeks | 20 | 21.3 (2.9) | 0.3 (-0.9 to 1.5) | 0.10 | 0.82 |  |
|  | 8 weeks | 19 | 19.8 (3.7) | -1.3 (-2.6 to 0.1) | -0.42 | 0.07 |  |
| **WHO Quality of Life (WHOQOL-BREF)** | Baseline | 21 | 82.5 (7.0) | - |  | - | 0.002 |
|  | 4 weeks | 20 | 84.5 (12.6) | 2.6 (-2.0 to 7.1) | 0.37 | 0.31 |  |
|  | 8 weeks | 19 | 88.4 (9.6) | 5.9 (2.2 to 9.7) | 0.84 | 0.003 |  |
| **Beck Depression Inventory (BDI)** | Baseline | 21 | 15.7 (5.7) |  |  |  | <0.0001 |
|  | 4 weeks | 19 | 13.8 (6.1) | -2.0 (-5.3 to 1.3) | -0.35 | 0.24 |  |
|  | 8 weeks | 19 | 9.4 (4.5) | -6.1 (-9.0 to –3.3) | -1.07 | 0.0002 |  |
| **Beck Anxiety Inventory (BAI)** | Baseline | 21 | 12.5 (6.0) | - |  | - | <0.0001 |
|  | 4 weeks | 20 | 11.9 (4.9) | -0.6 (-2.7 to 1.5) | -0.1 | 0.69 |  |
|  | 8 weeks | 19 | 7.5 (3.9) | -5.1 (-7.7 to -2.4) | -0.85 | 0.0006 |  |
| **Insomnia Severity Index (ISI)** | Baseline | 21 | 11.4 (5.5) | - |  | - | 0.003 |
|  | 4 weeks | 20 | 10.2 (5.3) | -1.2 (-3.3 to 0.7) | -0.22 | 0.23 |  |
|  | 8 weeks | 19 | 8.2 (5.3) | -3.5 (-5.5 to -1.4) | -0.64 | 0.002 |  |

*****P-value for mean change compared to the baseline (Dunnett`s adjustment method); repeated measures analysis of variance, intention to treat approach.

SD, Standard Deviation; CI, Confidence Interval; MBSR, Mindfulness-Based Stress Reduction
